# Just entertainment: effects of TV series about intrigue on young adults

**DOI:** 10.3389/fpsyg.2015.00529

**Published:** 2015-04-29

**Authors:** Fei Wang, Shengdong Lin, Xue Ke

**Affiliations:** ^1^Department of Advertising, School of Journalism and Communication, Xiamen University, Xiamen, China; ^2^School of Management, Xiamen University, Xiamen, China

**Keywords:** effects of media, intrigue, belief in justice, moral disengagements, interpersonal trust

## Abstract

The potential harmful effects of media violence have been studied systematically and extensively. However, very little attention has been devoted to the intrigue and struggles between people depicted in the mass media. A longitudinal randomized experimental group-control group, pretest–posttest design study was conducted to examine the potential effects of this type of TV series on young adults. A typical and popular TV series was select as a stimulus. By scrutinizing the outline of this TV series and inspired by studies of the effects of media violence, one behavioral observation and five scales were adopted as dependent measures. The study did not find any effect of the intrigue TV series on any of the six dependent variables. Finally, possible interference variables or moderators were discussed.

## Introduction

TV series about the intrigue and struggles in the Imperial Harem…undoubtedly have an adverse effect on the public social mentality…are misleading, especially for young adults…and make them escape from reality and indulge in cynicism.

*Comment in the People’s Daily (Wang, 2012)*

This comment on TV series about intrigue and struggles in the imperial harem came from *People’s Daily*, the most influential official newspaper in China. Therefore, this statement reflects the official ideology of the Chinese state and is also an example of popular wisdom, that is, that emphasizing the dark side of humanity in a TV series would destroy the ideals and values of the viewers, especially of young adults, and that TV series should provide a positive educational experience. However, an opposing position in popular wisdom holds that such comments are hyperreactive, that entertainment is entertainment and is not intended to be educational, and thus has no effect on viewers at all. “Give therefore to the emperor the things that are emperor’s, and to God the things that are God’s” (Holy Bible, 1995). Such contradictory arguments brought out the scientific detective in us. Do the conspiracies and intrigues in such a TV series influence the viewers’ beliefs or values and behavior in the real world? If so, what kinds of beliefs or values and behavior are influenced?

TV series which involved intrigue and struggle between people are often presented in Chinese television (Wang, 2012), and the plot usually turns on someone who plans a crafty and involves schemes to achieve his or her sinister ends. The court from several 100 years ago is generally the place for this intrigue in the TV series, but, actually, these stories are intended to reflect office politics in real life. Although these types of TV series attract many viewers and generate many arguments among folk scientists, Chinese academic research has provided surprisingly little specific empirical evidence on the effects of TV series about intrigue on the viewers’ beliefs or values and behavior. Fortunately, academic research has recognized the importance of the relationship between other specific content (such as violence and sexuality) in TV series and the viewers’ beliefs or values and behavior. These other studies inspired and served as references for the current study.

Violence as a pervasive social phenomenon has been extensively explored in various fields using a variety of methods. Some studies that employed longitudinal procedures showed that the more violent the television programs that participants watched as children, the higher their levels of aggression in the long run (Eron and Huesmann, 1980; Anderson and Bushman, 2002; Johnson et al., 2002; Huesmann et al., 2003; Gentile et al., 2004). Other researchers have used an experimental design to explore the short-term or long-term impact of exposure to media violence (video games, film clips, and TV violence) on undergraduates. All the results showed that media violence elicited aggressive attitudes, cognition, and behaviors (Kiewitz and Weaver, 2001; Anderson et al., 2004; Carnagey et al., 2007; Krahé et al., 2011). Meta-analysis studies further confirmed a causal relationship between viewing violence and these harmful effects (Eagly and Steffen, 1986; Wood et al., 1991; Paik and Comstock, 1994). All in all, the abundance of evidence converges on the conclusion that exposure to violent television significantly increases the likelihood of aggressive behavior of the people who were exposed to them in both immediate and long-term contexts. So, how does TV viewing affect the viewers’ behavior and beliefs or values?

### Routes through Which Mass Media May Influence the Viewer

How exposure to large amounts of specific (such as violent, sexual) content in television programs can influence a viewer’s beliefs or values and behavior has been explored extensively. Researchers have found at least three pathways that may explain this relationship:

The first is *perception bias*. The specific TV content may change an individual’s perception of the real world. Viewers who saw too many instances of violence on TV may exaggerate the frequency of violence in the real world, fear becoming a victim (Gerbner, 1994), have feelings of vulnerability (Reid and Finchilescu, 1995), and even have a strong expectation that others will behave aggressively (Bushman and Anderson, 2002). Sexual content may similarly influence viewers. People who view more sexual content are more likely to perceive their peers as sexually active and to develop sexually permissive attitudes and sexual relationships (Escobar-Chaves et al., 2005; Martino et al., 2005; Escobar-Chaves and Anderson, 2008).

The second pathway is *desensitization*. After regular or frequent exposure to violent content on TV, individuals become less sensitive to such materials and its consequences (Anderson et al., 2003). In contrast with individuals who do not view violent content, those who do showed less arousal to violent scenes (Bartholow et al., 2006; Krahé et al., 2011), more tolerance of real-life aggression (Drabman and Thomas, 1974; Molitor and Hirsch, 1994; Carnagey et al., 2007), less empathy toward others (Konrath et al., 2011), and enhanced aggressive thoughts and emotions (Funk et al., 2004).

The third path is *imitation*. Media portrayals also evoke *imitation*. Classic research developed by Bandura et al. (1963) clearly showed that children imitated aggressive behavior after they witnessed an aggressive adult model who attacked an inflated doll. In contrast, those children who were exposed to an adult model who just sat quietly in a room with an inflated doll did not show any aggressive actions. Children who were exposed to large numbers of sexual movies tended to experience early intercourse (Escobar-Chaves et al., 2005; Martino et al., 2005).

Mass media influence viewers by the same routes, whether the influence is violence or intrigue, so the paradigm for research about media violence can be adapted to research on TV series about intrigue and struggle. The effects of both short- and long-term exposure to media violence have been explored by using experimental, static observational, survey, and longitudinal designs (Paik and Comstock, 1994; Dill and Dill, 1999). The strengths and weaknesses of these methods were addressed in a study by Zillmann and Weaver (1999). Weighing the pros and cons of different methods and considering the purpose of the current study, a longitudinal design seemed to be the preferential choice. Furthermore, the properties of the stimuli helped to determine the experimental design. Intrigue in a TV series is more implicit than media violence, so the viewer needs to utilize a higher level of cognition in order to speculate about the intentions of the protagonist. Thus, children and adolescents are not their target viewers because they do not have the level of maturity necessary for comprehending the plot. The plot of an intrigue series is more complicated and unfolds much more slowly than violent plots. As a result, series which involve intrigue may have many episodes, so long-term exposure to the intrigue was necessary. For this combination of reasons, a longitudinal experiment was designed to address whether a TV series about intrigue and struggles between people could influence viewers’ beliefs or values and behavior.

### Beliefs, Values, and Behavior Which May be Influenced by TV Series

We should define what kind of belief or value and behavior that a TV series might influence before we go further to test whether they actually do. We will first outline the framework and key content of this type of TV series to identify the possible influences. Generally speaking, TV series about intrigue tell diverse stories, but actually have the same pattern. *The Legend of Zhen Huan* is typical of this genre. So, we took it as an example and an experimental stimulus.

*The Legend of Zhen Huan* is a Chinese ancient costume TV series which takes place in the Forbidden City during the Qing dynasty. It is so impactful that imitating the styles of expression of the characters has become fashionable and has even spread to the USA (Huang et al., 2013). This fictional story begins with how a naïve and innocent little girl survives in the cruel and harsh conflict between the empresses and concubines and eventually turns into a cunning and deceptive Empress Dowager (The Legend of Zhen Huan, 2013). It is of no surprise to anyone that the story has a happy ending, the good people get rewarded and the bad get punished. The utmost intrigue and scheming among the concubines of the emperor are repeated throughout the story. The concubines always try to cheat each other, betray each other, and struggle for power and status. After being exposed to such intensive cheating, betrayal, and brutal hurts to good people, the viewer might be expected to show a significant perception bias with respect to the world and society. They may exaggerate the pervasiveness of intrigue in the real world and doubt whether the world is just. Thus, we hypothesized that: after viewing the entire TV series of *The Legend of Zhen Huan*, the viewers’ general belief in a just world (GBJW) will decline (H1). Specifically, perception bias could be expected to make viewers fear becoming a victim of intrigue. So we postulated that: after viewing the entire TV series of *The Legend of Zhen Huan*, the viewers’ fear of becoming a victim of intrigue would increase, so their personal belief in a just world (PBJW) will decline (H2). Following the same logic, we further suggested that the viewing will damage interpersonal trust: after viewing the whole TV series of *The Legend of Zhen Huan*, viewers’ interpersonal trust will decline (H3). However, the TV series eventually has a happy ending, the bad guys get punished and the good guys get rewarded. Although injustices may occur in the world, they are all resolved and fairly compensated for in the end. So, we postulated that: after viewing the entire TV series of *The Legend of Zhen Huan*, viewers’ belief in ultimate justice (BUJ) will increase (H4). Desensitization to cheating and betrayal will make the viewer less sensitive to unethical conduct and likely to mitigate minor ethical code and norm violations in the context of daily transactions, so we postulated that: after viewing the entire TV series of *The Legend of Zhen Huan*, the desensitization mechanism will make the viewer more inclined to morally disengage (H5). Last, but not least, viewing the series will change not only the viewers’ beliefs or values but also their behavior. The characters’ performance may evoke imitative behavior in the viewers. Thus we postulated that: after viewing the entire TV series of *The Legend of Zhen Huan*, the viewer will show more dishonest behavior (H6).

Sex has also been examined in the literature as a potential moderator for the effect of media violence. Three meta-analytic reviews of gender differences (Eagly and Steffen, 1986; Wood et al., 1991; Paik and Comstock, 1994) showed that, although the predicted gender difference was found, the effect size was small and the finding was quite variable across studies. Some more recent research (Anderson et al., 2004; Krahé et al., 2011) also suggested that the effect was similar in men and women. In the same vein, no sex differences were anticipated in the current study.

## Materials and Methods

### Participants

The research was approved by the academic and ethics committee of the School of Journalism and Communication, Xiamen University. One hundred thirty-eight undergraduates who were enrolled in one course at Xiamen University participated in exchange for partial course credit. Their age ranged from 16.7 to 23.5 years (*M* = 20.0, SD = 1.1) with 84 being female (60.9%). Six participants were younger than 18 years old when they enrolled in this course, so we asked the political instructor who serves as the guardian for the undergraduates in Xiamen university to provide oral consent. All the participants had learned from the syllabus that they would view some TV series and complete two surveys if they enrolled in this course. They were also informed that they were free to discontinue participation at any time. All participants had learned from the syllabus that they would view some TV series and complete two surveys if they enrolled in this course, and they were also informed that they were free to discontinue participation at any time. Some students quit as the class proceeded, and some did not finish the second test, so the data obtained from these students were discarded.

### Procedure

This study was a longitudinal randomized experimental group-control group, pretest–posttest design. The participants were randomly assigned to one of two experimental conditions. About half of the students were asked to watch *The Legend of Zhen Huan* (a total of 76 episodes) over the course of a semester; the others were asked to watch any type of TV series of their choice. We did not choose a specific TV series for the control group because our plan was for the regular viewers would watch their own choice of a TV series in the general situation because the goal of this research was to contrast the effect of a “TV series with intrigue and struggle” with that of a “TV series without intrigue and struggle” in a general sense. To minimize the chances that the participants would detect the experimental hypothesis, the cover story stated that the tasks for the students were to find brand placements in the TV series and analyze the practice of the art of advertising. They were also asked to report the key plot of the TV series every 2 weeks to make sure they watched the full TV series. At the beginning of the semester, each student was requested to respond to a paper and pencil self-report scale battery. At the end of the semester (about 4 months later), they were asked to finish the scale battery again. Finally, the debriefing task asked the participants to speculate on the purpose of the study, after which they were dismissed.

### Variables and Measurement Instrument

#### Belief in a Just World

General and PBJW was assessed using BJW (Dalbert, 1999; Wu et al., 2011). It was measured by a 13-item scale comprising the six items of the GBJW Scale, which were used to assess whether the individual “believes in a just world where people generally get what they deserve” (Dalbert, 1999) and the seven items of the PBJW Scale (PBJW) which were used to assess whether the individual sees the world as just or unjust for himself (Lipkus et al., 1996; Dalbert, 1999). The responses were made on a 6-point scale ranging from 1 (strongly disagree) to 6 (strongly agree). The Cronbach’s α for the GBJW and PBJW subscales was 0.86 and 0.84, respectively, indicating good internal consistency (Wu et al., 2011).

#### Interpersonal Trust

The interpersonal trust (IT) scale was employed to assess “an expectancy held by an individual or a group that the word, promise, verbal or written statement of another individual or group can be relied upon” (Rotter, 1967, 1971). The participants were asked to agree or disagree (1 = strongly agree; 5 = strongly disagree) with a set of 25 statements. The item 14 “Most elected public officials are really sincere in their campaign promises” in the original scale was discarded from this study because it is not familiar to people in mainland China. In this study, the Cronbach’s α for the 24 item IT was 0.62.

#### Belief in Ultimate Justice

The BUJ scale was developed by Maes and Schmitt (1999) and Maes and Kals (2002) to measure “the tendency to believe that forthcoming events will settle any injustice that occurs.” The BUJ scale contains 13 items (for example: “Those who have suffered will be compensated 1 day”). Responses were given on a 6-point scale ranging from 1 (strongly disagree) to 6 (strongly agree). In this study, the Cronbach’s α for the BUJ scale was 0.90.

#### Civic Moral Disengagements

A 32-item civic moral disengagements (CMDs; Caprara et al., 2009; Wang and Yang, 2010) with a 5-point Likert scale (from 1 = agree not at all, to 5 = completely agree) response format was used to address the eight “psychosocial mechanisms by which individuals mitigate the moral consequences of harmful behaviors.” Four items corresponded to each of the eight mechanisms: Moral justification, euphemistic language, advantageous comparison, displacement of responsibility, diffusion of responsibility, distorting consequences, attribution of blame, and dehumanization. The Cronbach’s α of the 32-item CMDs for this study was 0.92.

#### Dishonest Behavior

Mazar et al. (2008) found that a low probability of being caught could increase the frequency and magnitude of dishonesty. Inspired by Mazar et al.’s (2008) work, we asked all the participants to finish a test with 30 multiple-choice, general-knowledge questions (e.g., “Which of these areas possesses rich water resources?” “What is the sun’s surface temperature in degrees?”). Both *The Legend of Zhen Huan* group and the other TV series group were further divided into two groups: participants who handed both the test and the answer sheet to the experimenter, who would check the correct number of answers, and a separate group of participants who only reported the number of correct responses to the experimenter, thus providing them with an opportunity to cheat. The participants were told in advance that they would be given different numbers of participation reward (file folders) based on the correct number of answers. The general-knowledge questions were completely different at the beginning of the semester from those at the end of the semester.

## Results

We checked the participants’ TV series watching every 2 weeks throughout the semester. The results showed that the experimental group continued to watch *The Legend of Zhen Huan* and the control group watched diverse types of TV series other than those TV series that involved struggle and intrigue. These included popular Chinese TV series (such as *Beijing Youth, iPARTMENT, Dwelling Narrowness*) and popular American TV series (such as *The Big Bang Theory, Two Broke Girls, and Friends*). This result demonstrated that our treatment manipulation was valid.

Because some students did not finish the second survey, the data obtained from these students were dropped. In the end, data from 127 participants were analyzed. Preliminary analyses [2 (gender) × 2 (type of TV series watched) analysis of covariance (ANCOVAs)] revealed no main or interaction effects of sex, so sex was dropped from further analyses.

The mean score and standard deviation of the five inventories can be seen in Table 1. Hypotheses 1–5 were tested through ANCOVA with the type of TV series watching as a between-subjects variable and the score on the five inventories before the TV series watching as a covariant variable.

**TABLE 1:** **Mean scores and standard deviations for GBJW, PBJW, IT, BUJ, CMDs by the testing time and the type of TV series watched**.

Variable	The Zhen Huan Legend (*N* = 62)		Other TV series (*N* = 65)	
	*M*	SD	*M*	SD
**GBJW**				
Before watching	4.05	0.84	3.97	0.77
After watching	4.21	0.82	4.10	0.73
**PBJW**				
Before watching	4.24	0.71	4.01	0.69
After watching	4.38	0.66	4.25	0.63
**IT**				
Before watching	3.11	0.32	3.06	0.31
After watching	3.14	0.32	3.05	0.30
**BUJ**				
Before watching	3.92	0.80	3.93	0.86
After watching	4.01	0.85	3.99	0.74
**CMDs**				
Before watching	2.13	0.45	2.15	0.43
After watching	2.14	0.43	2.19	0.42

Missing values were replaced by the mean scores for that column. GBJW, general belief in a just world scale; PBJW, personal belief in a just world scale; IT, interpersonal trust; BUJ, belief in ultimate justice; CMDs, civic moral disengagements.

The ANCOVAs indicated that there was no main effect of the type of TV series watching on a GBJW, *F*(1,124) = 0.32, *p* = 0.571; there was no main effect of the type of TV series watching on a PBJW, *F*(1,124) = 0.01, *p* = 1; there was no main effect of the type of TV series watching on IT, *F*(1,124) = 2.90, *p =* 0.091; there was no main effect of the type of TV series watching on a BUJ, *F*(1,124) = 0.04, *p* = 0.850; there was no main effect of the type of TV series watching on CMDs, *F*(1,124) = 0.19, *p* = 0.668, and there were no main effects of the type of TV series watching on any dimension of the CMDs, *p*s > 0.05. The scores on the five variables at the beginning of the semester significantly influenced the scores at the end of the semester, *p*s < 0.001, which means that, at least for these five measures, the opinion of the participants, regardless of whether they belonged to the experimental or the control group, remained consistent throughout the semester.

Therefore, hypotheses 1–5 were rejected. Thus, it appeared that a long term exposure to a TV series about intrigue did not change the viewers’ belief in justice or their IT and did not make them become more inclined to morally disengage.

### Impact on Dishonest Behavior

**FIGURE 1:**
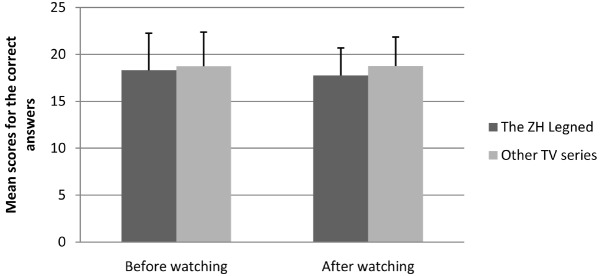
**Mean scores and standard deviations for the correct answers on the general knowledge (self-report) by the testing time and type of TV series watched**.

**FIGURE 2:**
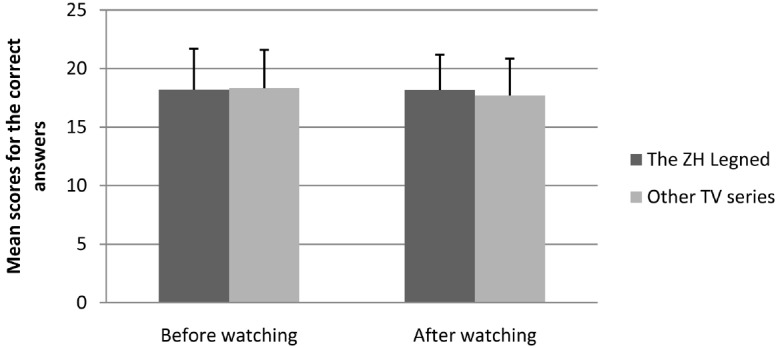
**Mean scores and standard deviations for the correct answers on the general knowledge (real score) by the testing time and type of TV series watched**.

Some student did not finish the final group of 30 general-knowledge questions, leaving 119 participants’ data to be analyzed further. The mean score and standard deviation of the general knowledge questions can be seen in Figures 1 and 2.

An analysis of variance (ANOVA) was conducted with the type of TV series watched (i.e., *The Zhen Huan Legend* vs. other TV series) and the method of scoring the general knowledge questions (i.e., self-report vs. real score) as between-subject variables and the score on the general knowledge (i.e., before watching the TV series vs. after watching the TV series) as a repeated-measures variable. We found no significant difference between the score for *The Zhen Huan Legend* group and that of the other TV series group, *F*(1,115) = 0.30, *p* = 0.571. Regardless of whether they had the chance to cheat (self-report or graded by the experimenter), they did not show dishonest behavior, *F*(1,115) = 0.34, *p* = 0.561. The three-way interaction was also not significant, *F*(1,115) = 0.81, *p* = 0.371, which means that *The Zhen Huan Legend* group and the other TV series group did not show any differences regardless of whether they had a chance to cheat and whether the test was taken after or before watching the TV series. Therefore, we rejected hypothesis 6.

## Discussion and Conclusion

The potential for harmful effects of mass media on viewers have been studied by social psychologists for many years. Especially with respect to media violence, the leading experts have come to an agreement: regular or frequent exposure to mass violence increases the likelihood of aggressive behavior by the viewer, and such effects are lasting and substantial (Anderson et al., 2003). Violence is not the only popular offering of the mass media; other kinds of “soft” violence, such as intrigue and struggles for power or status between people, are depicted in the mass media every day, especially in China. Do they influence the viewers in the same ways as media violence? Unfortunately, these effects have not been addressed extensively by academic research. This has allowed folk theorists to speculate and debate. Clearly, two conflicting arguments have been offered. The official ideology of the Chinese government, as reported in the official newspaper, is that these shows are a great scourge, but some viewers contend that such TV series are only entertainment and have no effect on them.

Before we began to address whether this type of TV series could influence the public social mentality, we first established the possible routes through which they might influence the viewer and what types of belief or value and behavior they might influence. We postulated that exposure to such extensive intrigues and struggles between people would change the viewers’ perception of justice, decrease their IT, make them morally disengage, and even cause them to decide to cheat. Hence, we conducted a longitudinal design in which, systematically over 4 months, some participants were exposed to a TV series which depicted intrigues and struggles between people whereas the others were exposed to other types of TV series. All the participants were measured with five well-validated and widely-used scales and one behavioral observation. Contrary to our hypotheses, we did not observe any differences between the experimental and the control group. That is, regardless of the type of TV series to which the viewers were exposed, their belief in justice, trust in other people and moral disengagement did not change throughout the semester. All six hypotheses were rejected. It seems reasonable to speculate that an intrigue-based TV series could influence viewers’ beliefs or values and behavior if it follows the same mechanism that causes media violence to influence viewers, but why did we not find any effect of the intrigue-based TV series on any of the six dependent variables? We speculate that a number of possible interference variables or moderators did not receive adequate attention. These factors fall roughly into three categories: the characteristics of the viewers, the characteristics of the intrigue-based TV series and the fitness of the dependent variables.

The viewers in this study were college students at an elite Chinese university. Obviously, their education level was not typical of the viewers of this TV series. Therefore, additional research should be done to address a possible moderating effect of education level. The age of the viewers was another possible moderator. Some research about the effects of media violence found that the effect was greater in a group of children than in a group of young adults (Anderson et al., 2003; but see Johnson et al., 2002). Our participants were mostly 20 year old college students, whose moral development level could have helped them easily distinguish right from wrong and enabled them to resist the temptation of the wrong model (Shaffer and Kipp, 2013). In addition, they may also have had many other interesting entertainment experiences during this same period. These two factors could have overridden or corrected the effects, if any, of the intrigue-based TV series on the young adults. The personality of the viewers is a third potential moderator. The concept of “reciprocal determinism” in social learning theory (Bandura, 1977) indicates that different types of viewers not only seek out different types of media content but also are affected differently by that content. Does a certain type of personality make viewers more apt to be attracted to and influenced by an intrigue-based TV series? This question deserves additional attention in future research.

The characteristics of the TV series’ content may have been another source of interference. The degree of realism, the similarity between the characters of this TV series and the participants in the study, and the portrayed justification of the intrigue should be thoroughly investigated. According to existing research about media violence, relatively realistic portrayals increase viewers’ aggression more strongly than relatively fictionalized portrayals (Geen, 1975; Atkin, 1983). Whether the aggressive character portrayed is similar to the viewer significantly impacts the effect of media violence (Bandura, 2001). In the current study, *The Legend of Zhen Huan* was used as the exposure material. This TV series portrayed brutal conflicts and struggles between concubines in the Qing dynasty. From the perspective of modern Chinese college students, the story took place in ancient China (300 years ago), which is a long time ago and a very different time. Thus, they may have perceived the intrigue content as unrealistic and did not find any similarity between the characters and themselves. However, the plots are simply a projection of office politics in real life. In other words, they may have perceived it as nothing but entertainment, but they may also have found more to relate to than would be obvious given the difference in era. Further studies will be needed to investigate these potential interferences. In order to be politically correct, this TV series did not portray the intrigues and struggles as justified; it depicted losses to both sides and in the end, the good were rewarded and the bad were punished. Friendship and love were also interspersed throughout the long story. Perhaps these themes mitigated the influence of the intrigue content. The characteristics of the media may have been another inference factor. Current research suggests that the interactive nature of many of the new media (such as video games) may have a more direct effect on viewers’ values and behavior than the more passive aspects of media such as TV (Anderson et al., 2003). Practicing intrigue behaviors, not just observing them, might have had a greater impact on the viewers’ values and behavior. The length of the exposure to the series may be another moderating factor. Short-term and more intense exposures to intrigue should be addressed in future studies. Last, but not least, the fitness of the dependent variable should be scrutinized. The reliability for the IT scale was 0.62, which is slightly lower than the recommended level of 0.70. This may limit both the reliability and validity of the study’s results. In connection with the dishonesty behavior manipulation, we used file folders as incentives. But these may have had too low a monetary value to incentivize cheating behavior, so future research should reconsider the value of the reward. Additionally, the sensitivity of this type of measurement for this particular situation is uncertain. We did not find any overt effect of the intrigue-based TV series, but we cannot be sure whether the intrigue-based TV series covertly influenced the viewers. Therefore, considering some implicit measures (Fazio and Olson, 2003; Gawronski et al., 2008), such as implicit association test (IAT; Greenwald et al., 2003), affect misattribution procedure (Payne et al., 2005), and semantic priming (Banaji and Hardin, 1996; Wittenbrink et al., 1997) should be helpful.

This study contributes to the literature on the effects of media and related areas by being the first study to assess the effects of a TV series about intrigue and struggle on young adults in a naturalistic setting with a longitudinal experimental manipulation. The study provided some evidence toward settling the controversies about whether TV series about intrigue have adverse effects on young adults. The results did not support the official ideology of the Chinese state and supported an opposing perspective of popular wisdom, which holds that TV series are nothing but entertainment, that is, that they do not “teach” viewers false values or induce them to participate in wrong behavior.

Although we found that exposure to media intrigue did not influence the viewers’ values and behavior in this study, the debate about the potential for adverse effects of media intrigue on the public social mentality is far from over. As we have discussed, too many potential interference variables or moderators remain unresolved to conclude that intrigue-based TV series have no effect on their viewers. All in all, more efficient manipulations, new paradigms, and more effective and sensitive measures should be considered in future research. For this type of study, our study is just a beginning.

### Conflict of Interest Statement

The authors declare that the research was conducted in the absence of any commercial or financial relationships that could be construed as a potential conflict of interest.
